# Association of age with left ventricular volumes, ejection fraction and concentricity: the Framingham heart study

**DOI:** 10.1186/1532-429X-15-S1-P264

**Published:** 2013-01-30

**Authors:** Michael L Chuang, Philimon Gona, Gilion Hautvast, Carol J Salton, Marcel Breeuwer, Christopher J O'Donnell, Warren J Manning

**Affiliations:** 1 NHLBI's Framingham Heart Study, Newton, MA, USA; 2 Cardiovascular Division, Beth Israel Deaconess Medical Center, Boston, MA, USA; 3 Imaging Systems, Philips Healthcare, Best, Netherlands

## Background

Left ventricular (LV) parameters may vary with aging, but concomitant disease can obscure relationships between age and LV size, mass and function. CMR provides reproducible measures of LV volumes, mass, and ejection fraction (EF), but manual analyses are time consuming. Automated border detection (ABD) methods may decrease analytical burden. We sought to examine the effect of greater age on LV volumes, mass, EF and concentricity in a large cohort of healthy adults free of clinical cardiovascular disease and hypertension using ABD-assisted methods.

## Methods

1494 members of the Framingham Heart Study Offspring cohort, serially followed since 1971, underwent cine SSFP CMR (TR/TE/FA=3.2 ms/1.6 ms/60deg; 10-mm thick contiguous short-axis slices) of the left ventricle at 1.5T. LV endo- and epicardial contours were automatically segmented by software (Extended MR Workspace 2009, Philips Healthcare), after which the operator had the option to adjust contours (if needed), followed by manual definition of the most basal slice at end-diastole. Concentricity was defined as LV mass/EDV (end-diastolic volume). We then identified a subset of healthy referent Offspring (N=685) free of any history of myocardial infarction, heart failure, CMR wall motion abnormality, and hypertension (SBP≥140 or DBP≥90 mmHg or antihypertensive medication use) and stratified by decade age (<50, 50-59, 60-69, ≥70 years). We compared sexes by 2-sample t test and used linear regression to test for within-sex trend across age groups. We assessed observer reproducibility (n=48) by intraclass correlation coefficient (ICC).

## Results

Referent participants were aged 61±9 years (262 men, 423 women). Men had greater LV volumes, mass and concentricity than women (p<0.0001, all), but women had greater EF (73±6 vs. 71±6%, p=0.0002). As the Table (mean±SD) shows, LV EDV, end-systolic volume (ESV), and stroke volume (SV) decreased with greater age in both sexes; LV mass decreased with age in women but not men. After indexation to body surface area (BSA) trends in volumes remained, but mass/BSA did not trend with age in either sex. Both LV EF and concentricity increased with age in both sexes. ABD-assisted analysis was highly reproducible with interobserver ICC=0.99, 0.98 for EDV and ESV, 0.99 for LV mass, and 0.96 for EF.

**Table 1:** 

	P (trend)	<50 y	50-59 y	60-69 y	≥70 y
MEN, N		26	104	94	38

EDV, ml	<0.0001, ↓	168±28	155±27	145±29	136±28

ESV, ml	<0.0001, ↓	53±16	47±13	41±14	37±10

SV, ml	0.005, ↓	109±18	109±19	103±20	99±21

Mass, g	0.36	94±13	103±20	96±22	97±20

EDV/BSA, ml/m^2^	0.0001, ↓	79±12	76±13	71±14	69±14

ESV/BSA, ml/m^2^	<0.0001, ↓	26±7	23±6	21±7	19±5

SV/BSA, ml/m^2^	0.03, ↓	54±9	53±9	51±9	50±11

Mass/BSA, g/m^2^	0.73	46±5	50±9	47±9	49±9

Mass/EDV	0.0036, ↑	0.77±0.12	0.84±0.17	0.85±0.16	0.92±0.30

EF, %	<0.0001, ↑	68±6	70±6	72±6	73±5

					

Women, N		24	179	139	81

EDV, ml	<0.0001, ↓	127±22	116±21	108±19	103±17

ESV, ml	<0.0001, ↓	38±10	33±9	30±9	25±7

SV, ml	<0.0001, ↓	89±17	83±16	78±13	78±13

Mass, g	0.009, ↓	68±18	59±13	56±11	28±11

EDV/BSA, ml/m^2^	<0.0001, ↓	69±8	66±10	62±9	61±9

ESV/BSA, ml/m^2^	<0.0001, ↓	21±5	19±5	17±5	15±4

SV/BSA, ml/m^2^	0.02, ↓	48±6	47±8	45±7	46±7

Mass/BSA, g/m^2^	0.27	37±7	34±6	33±5	34±6

Mass/EDV	<0.0001, ↑	0.70±0.09	0.72±0.86	0.74±0.11	0.78±0.12

EF, %	<0.0001, ↑	70±6	71±6	73±5	76±5

## Conclusions

In a cohort of adults strictly free of clinical cardiovascular disease and hypertension, LV volumes, but not mass, decreased with greater age. LV concentricity and ejection fraction increased with age. Whether the increase in concentricity represents normal healthy aging or subclinical disease remains to be investigated.

## Funding

Supported in part by NIH grant RO1 AG17509 and subcontract N01-HC-38038, and by an unrestricted educational grant from Philips Healthcare.

